# Can an intervention based on a serious videogame prior to cognitive behavioral therapy be helpful in bulimia nervosa? A clinical case study

**DOI:** 10.3389/fpsyg.2015.00982

**Published:** 2015-07-14

**Authors:** Cristina Giner-Bartolomé, Ana B. Fagundo, Isabel Sánchez, Susana Jiménez-Murcia, Juan J. Santamaría, Robert Ladouceur, José M. Menchón, Fernando Fernández-Aranda

**Affiliations:** ^1^Department of Psychiatry, Bellvitge University Hospital-IDIBELL, Barcelona, Spain; ^2^Ciber Fisiopatologia Obesidad y Nutrición, Instituto de Salud Carlos III, Barcelona, Spain; ^3^Clinical Sciences Department, School of Medicine, University of Barcelona, Barcelona, Spain; ^4^Université Laval, Québec, QC, Canada; ^5^Ciber Salud Mental, Instituto de Salud Carlos III, Barcelona, Spain

**Keywords:** eating disorders, bulimia nervosa, impulsivity, serious game, A-B-A-C-A design

## Abstract

**Background:** Several studies have highlighted the implications of impulsivity and novelty seeking for both the maintenance and the process of recovery from bulimia nervosa (BN). Cognitive behavioral therapy (CBT) is the treatment of choice for BN, but for some cases, this treatment alone might not be sufficient for reducing the high levels of impulsivity. The paper presents a case report of a patient with BN, examining the effectiveness of using a videogame (VG; Playmancer) as an additional intervention designed to address impulsivity.

**Design:** Psychometric and neuropsychological measures were collected at baseline. After this assessment, Playmancer was applied prior to CBT, following an “A-B-A-C-A” single case experimental design. Impulsivity levels were assessed with the Conner’s Continuous Performance Test II (CPT-II). After the Playmancer treatment, the patient started CBT, and the levels of impulsivity were recorded again. Finally, psychometric and neuropsychological measures were collected after treatment. Weekly frequency of binges and vomiting were also recorded during the entire procedure.

**Results:** After the VG intervention, psychometric measures such as anxiety levels, impulsivity and novelty seeking decreased. Regarding the neuropsychological measures, impulsivity levels (measured with the CPT-II) progressively decreased throughout the intervention, and an improvement in decision making capacities was observed. Furthermore, the frequency of binges also decreased during and after the VG intervention.

**Discussion:** This case report suggests that using the Playmancer VG to reduce impulsivity prior to CBT may enhance the final results of the treatment for BN.

## Introduction

Bulimia nervosa (BN) has often been associated with impulsivity (Fischer et al., 2003; Vaz-Leal et al., 2014). As already seen in other studies on mental disorders, as in addictions and personality disorders (Barnicot et al., 2011; Brorson et al., 2013; López-Torrecillas et al., 2014), the behavioral correlates of impulsivity, such as disinhibition, impulsive decision making and emotional dysregulation, are predictive factors in the prognosis of the treatment (relapse and dropout rates). Therefore, impulsivity must be taken into account in the treatment of eating disorders (ED), particularly due to its relevance as triggering and maintaining factor of ED symptomatology (Fischer et al., 2003; Vaz-Leal et al., 2014; Wenzel et al., 2014).

Several studies have pointed out the implications of impulsivity and novelty seeking in the recovery from BN, being associated to a higher risk of therapy failure, a lower remission rate and dropout (Agras et al., 2000; Walsh et al., 2000; Fassino et al., 2003a,b; Fernandez-Aranda et al., 2009; Castellini et al., 2012; Halmi, 2013). Though cognitive behavioral therapy (CBT) is the treatment of choice for BN (Fairburn et al., 1993; Wilson and Shafran, 2005; Shapiro et al., 2007), in some cases, this treatment modality alone might be insufficient for reducing high levels of impulsivity (Agüera et al., 2012; Hedman et al., 2014). Some studies have evaluated the effects of CBT on measures of temperament and character across treatment (Anderson et al., 2002; Agüera et al., 2012). The results suggest that, while some traits such as self-directedness and harm avoidance are susceptible to change after CBT, one of the traits that appears to remain stable is novelty seeking (strongly associated with impulsivity). CBT for BN is aimed at treating issues such as eating symptomatology (binge episodes and compensatory behaviors), cognitive restructuring, self-esteem and body image (Fairburn et al., 1993), but does not address impulsivity *per se*. Moreover, impulsivity is associated with a temperament dimension of personality that is less susceptible to change (Cloninger et al., 1993; Agüera et al., 2012). For these reasons, it is not surprising that impulsivity is not altered after CBT, at least not without introducing an additional intervention specifically designed to treat it.

Given the strong association between BN and impulsivity, as well as the influence of the latter on both the adherence to and efficacy of the treatment, additional intervention strategies need to be explored and evaluated. In the ED field, some studies have evaluated the effectiveness of combining CBT with a mindfulness-based intervention in order to enhance emotional regulatory strategies and improve the therapeutic outcome of women with binge eating disorder (BED). The results showed that the use of this additional intervention based on meditation techniques improved the awareness and acceptance of bodily signals (i.e., signals of hunger and satiety), and favored both conscious food choices and emotional self-regulation (Kristeller and Hallett, 1999; Kristeller and Wolever, 2011; Woolhouse et al., 2012). This was reflected in the reduction of both the number and intensity of binge eating, the improvement of the attitudes toward food and the decreased body image dissatisfaction as well as the levels of depression and anxiety (Kristeller and Hallett, 1999; Kristeller and Wolever, 2011; Woolhouse et al., 2012).

Another intervention applied in the field of ED, particularly in anorexia nervosa (AN), is Cognitive Remediation Therapy (Abbate-Daga et al., 2012; Davies et al., 2012; Pretorius et al., 2012). These patients have problems with “cold” cognition, which refers to that based on logic and rational thinking, and “hot” cognition, which refers to that based on intuition, emotional response and motivation (Schmidt and Treasure, 2006; Chan et al., 2008; Davies et al., 2012). However, treatment as usual (TAU) for AN does not focus on addressing these issues. In a study of Davies et al. (2012), the intervention strategy known as Cognitive Remediation and Emotion Skills Training (Money et al., 2011; Tchanturia et al., 2014), which addresses these difficulties, was applied to AN patients in addition to TAU to improve the therapeutic outcome. The intervention aimed to promote issues such as emotional expression and to target rigid and detail-focused thinking styles typical of AN patients (Davies et al., 2012).

Motivational Enhancement Therapy is another intervention that has been applied as a pretreatment or simultaneous to the TAU for ED, specifically for AN, BN, and ED not otherwise specified. The main goal is to address the patient’s ambivalence to change (Feld et al., 2000; Dean et al., 2008). Motivational Enhancement Therapy uses motivational techniques, such as a decisional balance exercise on the benefits and costs of having an ED and a discussion on the patient’s life values and goals in relation to the ED (Feld et al., 2000). Some of the benefits that have been reported after this type of intervention not only include an improvement in the motivation toward treatment, but also a decrease in the depressive symptoms and interpersonal distrust of the patient, as well as an increase in self-esteem (Feld et al., 2000).

To address impulsiveness, which is the factor of interest in the current study, some studies have demonstrated the efficacy of biofeedback interventions to treat impulse control difficulties in some mental disorders such as personality disorders and the attention-deficit/hyperactivity disorder. Howard et al. (2013) used biofeedback techniques to reduce impulsivity and inattention in a group of men with borderline, antisocial and histrionic personality disorders. The results showed a reduction of impulsivity and an improvement in attention, suggesting that this intervention strategy may be useful for greater behavioral and emotional self-regulation in these patients. Cho et al. (2004) showed that the effectiveness of neurofeedback combined with a virtual reality environment to improve levels of impulsiveness and inattention in people with social problems. Similarly, in a systematic review about neurofeedback in attention-deficit/hyperactivity disorder, Arns et al. (2014) concluded that neurofeedback could be a clinically effective treatment for children with this disorder.

Despite the existence of tools based on biofeedback techniques that are employed to treat impulsivity across various mental disorders (Schoenberg and David, 2014), little is known about their adequacy in the treatment of ED (Bartholdy et al., 2013; Scolnick et al., 2014).

Virtual reality is another additional technique employed in the field of ED, primarily used in the treatment of body image and to enable exposure to food in virtual environments (Riva, 2011; Cesa et al., 2013; Marco et al., 2013; Perpiñá et al., 2013). However, little is known about its usefulness in impulsivity and emotion regulation (Rodríguez et al., 2012; Cristea et al., 2014). Videogames (VG) can also be used in combination with TAU as complementary tools in the clinical field. These are referred to as “serious games.” Contrary to conventional VG, serious games are games designed for a specific purpose that goes further than pure entertainment. For example, they can be applied to improve the individual’s skills, attitudes, knowledge, etc (Djaouti et al., 2011; Graafland et al., 2014; Granic et al., 2014). This type of VG has been employed in health care with varying clinical goals among which are: obesity prevention and intervention (Lu et al., 2013), behavioral improvements in adolescents and young adults with cancer (Kato et al., 2008), the assessment and rehabilitation of elderly people with mild cognitive impairment, Alzheimer’s disease, and related disorders (Manera et al., 2015), and the prevention of alcohol and drug use in adolescents (Rodriguez et al., 2014). Yet, once again, there are few studies that explore the usefulness of serious games in the field of ED and emotion regulation (Fernandez-Aranda et al., 2012; Rodríguez et al., 2012).

It is possible to conclude that most of additional intervention strategies used in the field of ED have not addressed impulsivity. Moreover, these additional interventions have been applied simultaneous to the TAU, not as a pretreatment intervention. The application of an additional intervention prior to TAU might result in the improvement of specific skills that might be beneficial to the outcome of TAU.

This paper presents a case report of a patient diagnosed with BN, using a VG based on biofeedback (Playmancer; Jimenez-Murcia et al., 2009; Fernandez-Aranda et al., 2012) as an additional intervention to TAU (CBT) designed to address impulsivity control and emotion regulation. This tool was used prior to TAU. In order to evaluate the sequence of the intervention a single case experimental design “A-B-A-C-A” was used (Barlow and Hersen, 1984).

## Case Report

The study was carried out in the Eating Disorders Unit of the University Hospital of Bellvitge in Barcelona (Spain). The patient was informed about the study and signed the corresponding informed consent document, agreeing to participate in the study. The Ethical Committee of our institution approved the procedures.

The participant, a 34 year-old woman, married and mother of two children, came to our unit seeking treatment for her eating disturbances. She had suffered from substance abuse until she was 21 years old, age at which she received psychological treatment for mood problems. The patient reported an increase in weight from this age, going from a body mass index (BMI) of 21.3–27.7 Kg/m^2^. At the age of 27, she had her first pregnancy, accompanied by a new weight gain reaching a BMI of 31.9 Kg/m^2^. After her second pregnancy, at the age of 32, an additional increase of weight took place (BMI 34.0 Kg/m^2^). At the time of inclusion in the study, the patient had a BMI of 31.6 kg/m^2^, and she reported the presence of daily binges during the previous 3 months, accompanied by a great feeling of hopelessness and guilt, and afterward with compensatory vomiting episodes. According to DSM-5 diagnostic criteria (American Psychiatric Association, 2013), the patient was diagnosed with BN.

During the first interview, the patient also presented some traits of the Cluster B personality disorders of the DSM-5 (American Psychiatric Association, 2013), characterized by high impulsivity, low tolerance to frustration and poor emotional regulation. Occasional episodes of excessive buying were also present. Moreover, the patient was undertaking a pharmacological treatment with an antidepressant (Fluoxetine 20 mg: 1-1-0) since 6 months prior to the intervention. This medication plan remained stable during the entire treatment procedure.

## Assessment

### Neuropsychological Measures

The neuropsychological tests were selected to cover various aspects of executive functions including impulsivity and decision making. The patient was assessed with the following neuropsychological tests:

*CPT-II-Conner’s Continuous Performance Test II* (Conners and Staff, 2000): This is a classical sustained attention and impulsivity test. Subjects have to respond by pressing a button to target letters presented on the computer screen except when the letter “X” appears. In the present study we used the number of commission errors (when a response is given after an “X” letter appears on the screen), which is the most important measure of impulsivity. For the commissions subtest a split-half reliability of *r* = 0.83 and retest reliability of *r* = 0.65 have been obtained. In addition, the test has been shown to be relatively unaffected by practice effects (Conners and Staff, 2000). For the purpose of the study this is one of the main dependent variables.

*Iowa Gambling Task* (IGT; Bechara et al., 1997): This computer task evaluates decision making, risk and reward and punishment values. Higher results indicate better performance.

### Eating Symptomatology

The eating and purging symptoms of the patient (weekly frequency of binging and vomiting) were recorded by means of food diaries elaborated by the patient. For the purpose of the study this is the other main dependent variable (in particular the weekly frequency of binging).

### Psychometric Measures

When the patient came to our Unit for the first visit, she was assessed via a structured clinical interview for the DSM-IV Axis I disorders (SCID-I; First et al., 1996). For the rest of the psychological assessment, commonly applied questionnaires in the field of ED were employed:

*Eating Disorder Inventory 2* (EDI-2; Garner, 1991): This is a reliable and valid 91-item multidimensional self-report questionnaire that assesses different cognitive and behavioral characteristics, which are typical of ED. This instrument was validated in a Spanish population with a mean internal consistency of 0.63 (coefficient alpha; Garner, 1998).

*Temperament and Character Inventory-Revised* (TCI-R; Cloninger, 1999): This is a 240-item, reliable and valid questionnaire that measures seven dimensions of personality. The performance on the Spanish version of the original questionnaire (Gutierrez et al., 2001) and the revised version (Gutiérrez-Zotes et al., 2004) has been documented. The scales in the latter showed an internal consistency (coefficient alpha) of 0.87.

*Symptom Check List-90 items-Revised* (SCL-90-R; Derogatis, 1990): This is a 90-item multidimensional self-report questionnaire assessing a broad range of psychological problems/symptoms. This questionnaire has been extensively validated in a Spanish population, obtaining adequate psychometric values (Martinez-Azumendi et al., 2001). The Symptom Checklist-Revised-90-Revised has been validated in Spanish and has been previously described (Derogatis, 2002).

*State-Trait Anxiety Inventory* (STAI-S-T; Spielberger et al., 1970): This is a self-report questionnaire that includes 40 items. It evaluates the temporary condition of “state anxiety”(S) (20 items) and the more long-standing quality of “trait anxiety” (T) (20 items). The psychometric studies in a Spanish population achieved good reliability indices (Guillen-Riquelme and Buela-Casal, 2011). The STAI has been validated in Spanish and have been previously described (Spielberger et al., 1982).

*Barratt Impulsiveness Scale-version 11* (BIS-11; Patton et al., 1995; Oquendo et al., 2001): This is a 30-item self-report instrument designed to assess the multidimensional personality construct of impulsivity.

## Intervention Strategies

### Videogame Intervention

The Playmancer VG is a serious game that was used as an additional therapeutic tool, combined with standard psychological approaches (Jimenez-Murcia et al., 2009; Fernandez-Aranda et al., 2012). The main goal of this intervention is to improve self-control, to reduce impulsivity and to learn how to regulate both emotions (such as frustration and anxiety) and physiological reactivity when the player is confronted with several challenges. This is achieved via the analysis of biosensor and facial expression assessment measures (Claes et al., 2012), which are measured during the entire process. These instruments are used to provide biofeedback in order to train emotional regulation. Each session consists of the exposure to the VG, while the performance of patients is collected during 20 min. Relaxing music is played for 3 min before and after the VG. The level of game difficulty is adjusted in a closed feedback loop; higher levels of undesired emotional and/or physiological reactions are coupled with greater difficulty in attaining the end goals. Playmancer is an adventure-simulation game that takes place in a particular island of an archipelago where an avatar (the patient) is faced with different challenges and situations. The VG is composed of three activities (or mini-games). The first is “The Face of Cronos,” in which the patient has to plan a path to climb up a reef, avoiding certain obstacles that appear based on the patient’s arousal levels. The main goal is to train planning, decision making and self control abilities. In the second, “Treasures of the Sea,” the patient has to dive under the sea in order to find different treasures, taking into account that they need to control the oxygen consumption (higher arousal produces more oxygen expenditure). In this mini-game, the patient learns to train visuospatial and problem-solving abilities, as well as self control. Finally, “Signs of the Magupta” entails the patient connecting stars to complete a constellation by regulating their breathing. This mini-game is applied to train relaxation abilities and increase self awareness. More information about the VG system is available in the following link:

https://www.youtube.com/watch?v=osmo9EAClv8.

### Cognitive Behavioral Therapy Intervention

This intervention is an outpatient group therapy based on the model of Fairburn et al. (1993). The treatment consists of 16 weekly outpatient sessions (90 min each) with a total of 8–10 patients per group. The group is conducted by an experienced psychologist and a co-therapist. As described by Fairburn et al. (1993), one of the main goals of this group is to eliminate binge episodes and compensatory behaviors, as well as to establish correct nutritional patterns. This program focuses on issues such as rationale of cognitive model, training in problem solving strategies and cognitive restructuring, and addresses self-esteem, body image, body weight and relapse prevention strategies. This program and accompanying material have already been manualized and published in Spanish (Fernandez-Aranda and Turon, 1998) with demonstrated effectiveness (Fernandez-Aranda et al., 2004; Agüera et al., 2013).

## Study Design

**FIGURE 1:**
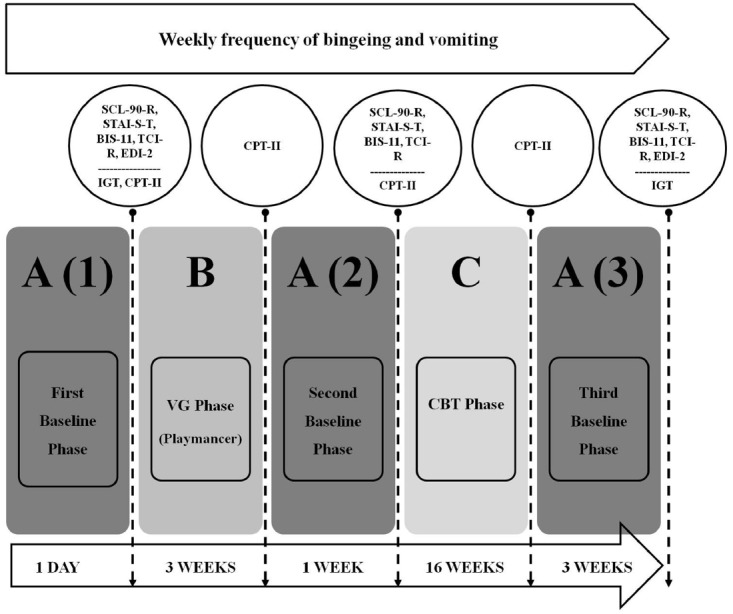
**“A-B-A-C-A” design**.

The experimental design that was used in this study followed an “A-B-A-C-A” model. The different phases of the design as well as the measures collected are presented in Figure 1.

### First Baseline Phase: A (1)

This first stage of the design consisted in obtaining initial psychometric and neuropsychological information of the patient prior to any intervention, with particular emphasis on impulsivity levels measured using the CPT-II. As explained in the method section, the CPT-II performance is one of the main dependent variables of the study.

### Videogame Phase: B

In this second phase, the use of a therapeutic VG (Playmancer) prior to TAU (CBT) for BN was performed. The duration was 3 weeks, during which nine sessions of 26 min were conducted (3 min of relaxing music, 20 min of VG and 3 min of relaxing music again). At the end of this phase, we re-analyzed the levels of impulsivity using the CPT-II, in order to assess whether there was any change in this measure after this preliminary intervention.

### Second Baseline Phase: A (2)

At the end of the waiting period before starting CBT (i.e., 1 week after finishing the first intervention with the VG), the psychometric variables and the levels of impulsivity (evaluated with CPT-II) were collected again. The aim of this phase was to analyze whether the expected changes were maintained after the VG intervention.

### Cognitive Behavioral Therapy Phase: C

In this phase, the patient started the TAU for BN: group CBT. During the treatment, the levels of impulsivity measured by the CPT-II were re-analyzed. The objective of this phase was to assess the evolution of the impulsivity after a standard intervention.

### Third Baseline Phase: A (3)

In this phase, another evaluation took place 3 weeks after completing CBT. The neuropsychological and psychometric data were explored again. The objective of this final phase was to analyze the changes in these measures after completion of all the therapeutic process.

During each of the above phases, the eating-related symptomatology of the patient (weekly frequency of binging and vomiting) was also recorded. As explained in the method section, this is one of the main dependent variables of the study (in particular the weekly frequency of binging).

## Results

The evolution of the main dependent variables of the study along the different phases is described below.

### First Baseline Phase: A (1)

Of the CPT-II test, the number of commission errors were used, which is the most relevant measure of impulsivity. The results showed a mildly atypical score of 19. In relation to the decision making capacities, the patient obtained a typical score in the low-average range in the IGT (NET Total Score: 8). As for the other main dependent variable, which concerns the eating symptomatology (frequency of binging), the patient showed a weekly average of 14 binges in this phase.

### Videogame Phase: B

At the end of this phase the patient obtained a mildly atypical score of 16 in the CPT-II. Regarding the eating symptomatology, a reduction in the frequency of binging was observed, whereby the average frequency of the binge episodes over the 3 weeks of VG was 7.7.

### Second Baseline Phase: A (2)

In this phase the patient obtained a mildly atypical score of 17 in the CPT-II. Moreover, the weekly average of binges was reduced to 2.

### Cognitive Behavioral Therapy Phase: C

In this phase the patient showed again a reduction in commission errors of the CPT-II, obtaining a score within the average range of 14. Regarding the frequency of binging, it continued declining, reaching a weekly average of 0.7.

### Third Baseline Phase: A (3)

In terms of the decision making capacities, an improvement was observed in the IGT scores in comparison to the scores at the beginning of the procedure. The patient was more likely to select the advantageous cards in this second administration than in the first assessment, shifting from a typical score in the low-average range in the first administration to one in the high-average range (NET Total Score: 56) in the second. These results suggest an improvement at treatment end in terms of the decision making capacities and the ability to analyze the possible consequences of a certain choice. Finally, the frequency of binging had decreased to 0 after the completion of the CBT.

**FIGURE 2:**
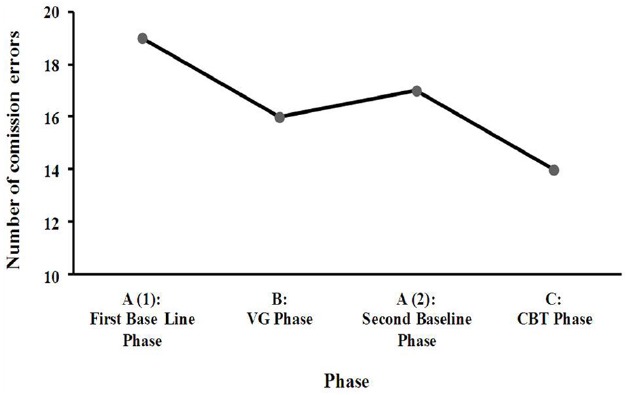
**Number of commission errors (CPT-II) across the different phases**.

**FIGURE 3:**
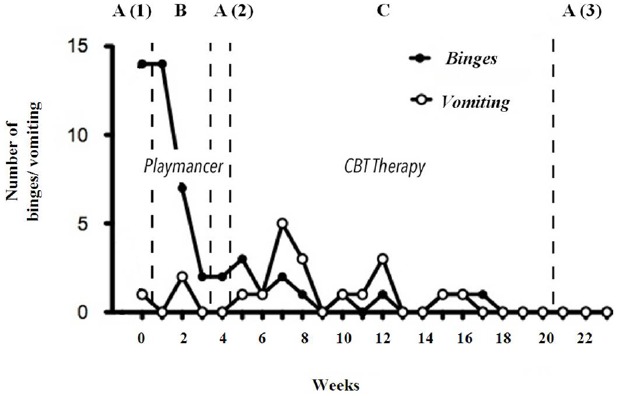
**Treatment of VG before CBT and influence on binge/vomiting episodes**.

Therefore, regarding the neuropsychological variables of the present study, the results showed that there were notable changes in impulsivity levels (measured with the commission errors of the CPT-II) across the different phases (Figure 2), as well as in the decision making, risk, reward and punishment values (measured with the IGT). In relation to eating symptomatology, a significant reduction in the weekly frequency of binge eating was observed during the phases prior to CBT (i.e., during the VG phase and Second baseline phase). In addition, the number of binges continued to show a downward trend throughout the remaining phases. With respect to the evolution of the frequency of vomiting, although it was more irregular, there was also a decreasing trend (Figure 3).

### Psychometric Measures

Regarding the main psychometric measures (Table 1), in the First baseline phase the typical profile of BN was observed, characterized by elevated symptoms of depression, anxiety (both state and trait), somatization and motor impulsiveness, as well as high novelty seeking (a personality trait associated with impulsivity). In the Second baseline phase, which takes place after the VG phase and prior to any other intervention, a further decrease in the aforementioned measures was found. Finally, in the Third baseline phase, shortly after the completion of CBT, there was a notable further reduction in the depressive symptoms, the levels of anxiety (both state and trait), and motor impulsivity. Regarding the ED psychopathology, as measured with the EDI-2 total score, a clear improvement was observed when comparing the First and Third baseline scores (107 and 38 respectively).

**TABLE 1:** **Main psychometric results across the different phases**.

	**First**	**Second**	**Third**
	**baseline**	**baseline**	**baseline**
	**phase A (1)**	**phase A (2)**	**phase A (3)**
Depression (SCL-90-R)	2,92	2	1,69
Somatization (SCL-90-R)	3,17	2,66	0,25
State anxiety (STAI-S-T)	46	36	28
Trait anxiety (STAI-S-T)	35	27	23
Motor impulsiveness (BIS-11)	27	25	23
Novelty seeking (TCI-R)	130	123	127

## Discussion

This case study aimed to assess the efficacy of a therapeutic VG (Playmancer), which addresses impulsivity, as a complementary tool in the treatment of BN. At the end of the treatment, the patient presented lower novelty seeking, fewer commission errors, an improvement in decision making capacity, and a decrease in the frequency of the binge eating episodes.

Impulsivity is a trait that has often been associated with deficits in decision-making, which is an important factor when studying the mechanisms that underlie the disadvantageous risky choices made in tasks such as in the IGT (Zermatten et al., 2005; Franken et al., 2008; Upton et al., 2012; Ochoa et al., 2013). Decision making is a cognitive function that consists in taking into account the consequences of a particular option before making a choice. Therefore, in the case of the IGT, a good performance implies a tendency toward choosing the safe decks, which provide a profit in the long run despite producing small short-term gains (Clark et al., 2004). Differently, impulsive behavior is characterized by a preference for the risky decks, which give fewer and higher short-term gains but produce elevated long-term losses, rather than aiming for greater benefits in the long run. Therefore, those individuals that are more impulsive tend to think less about the consequences of their choices (Franken et al., 2008). In IGT, higher levels of impulsivity, or impulsive decision- making, are reflected in the lower total scores (NET Score; Franken et al., 2008).

Regarding the patient of this single case study, the results of the IGT showed improvements in decision making at the completion of the treatment process. This may be further evidence for the reduced levels of impulsivity indicated by both psychometric (e.g., lower novelty seeking) and other neurocognitive measures (less commission errors in the CPT-II) after the treatment procedure.

Another important aspect of the results regards the patient’s eating symptomatology. At the completion of the first intervention (the therapeutic VG) a notable reduction in the frequency of binges was observed. Given that binges are a clear example of impulsive behavior, their decrease after the VG intervention can be interpreted as further evidence of the declining impulsivity levels already achieved by the patient prior to the start of the TAU (CBT).

Finally, the patient also reported a highly positive subjective impression in terms of the VG. She reported that one of the learned strategies that she applied most in her day to day life was relaxation trough breathing regulation. Specifically, in those situations in which she felt more anxious, she tried to imagine the constellations mini-game (“Signs of the Magupta”) in order to breathe slowly and deeply and thus reduce her anxiety. This is an important factor to consider as it shows the extrapolation of the knowledge acquired with the VG to real life.

However, the results of the present study must be interpreted in the context of certain limitations. First, there is a lack of a control subject. Second, being a single case study, the results cannot be generalized. Future studies should replicate this study with a larger sample, including a control group, as well as explore different designs. Third, the patient was undertaking a pharmacological treatment with an antidepressant (Fluoxetine 20 mg) during the procedure. However, this type of medication plan is very common in the clinical population and in the case of our patient it remained unchanged during the entire treatment procedure. Furthermore, given that the impulsivity levels of the patient were high at the beginning of the study even though the patient had been taking the antidepressant for 6 months prior to the start of the procedure, it is probable that the medication had a low influence on her impulsivity and control capacities. Despite these limitations, this study also has several strengths. First, it employs a novel therapeutic technique based on a VG intervention. Secondly, the VG is primarily intended to control impulsivity, which is an aspect that is not often directly addressed in most therapeutic interventions. Last, this study applies an innovative “A-B-A-C-A” design, which differs from the standard A-B-A models in which the efficacy of a treatment is evaluated only with pre and post measures.

In conclusion, in this case study the use of the Playmancer VG was an intervention strategy that successfully reduced impulsivity and improved decision making capacities. In addition, the VG intervention was also beneficial in order to reduce ED symptomatology. As described above, impulsivity is a maintaining factor of BN and a predictor of a poor response to treatment. However, this factor is barely addressed in CBT. Therefore, the integration of this VG intervention to reduce impulsivity prior to starting TAU may enhance the final results of CBT.

## Author Contributions

All authors designed the work and revised it for important intellectual content. CG, AF, FF, IS, and JS collaborated in the collection and the interpretation of the data. CG, FF, and AF drafted the study. All authors revised, commented on and approved the final manuscript and are accountable for all aspects of the work.

### Conflict of Interest Statement

The authors declare that the research was conducted in the absence of any commercial or financial relationships that could be construed as a potential conflict of interest.
